# Chemoprevention in Skin Cancer: What Advice?

**DOI:** 10.3390/cancers18030436

**Published:** 2026-01-29

**Authors:** Ariadna Ortiz-Brugués, Carmen Orte Cano, Lluis Corbella, Francesc Alamon-Reig, Ignasi Martí-Martí, Maria Ayguasanosa-Avila, Marc Hernández-Santacana, Priscila Giavedoni, Paula Aguilera, Cristina Carrera

**Affiliations:** 1 Hospital Clínic Barcelona, 08036 Barcelona, Spainimartim@clinic.cat (I.M.-M.);; 2 Department of Dermatology, Hôpital Erasme, Université Libre de Bruxelles (ULB), HUB, 1070 Brussels, Belgium

**Keywords:** chemoprevention, skin cancer, non-melanoma skin cancer, actinic keratosis, melanoma, solid organ transplant recipients, nicotinamide, retinoids, 5-fluorouracil, photodynamic therapy

## Abstract

The incidence of melanoma and non-melanoma skin cancers (NMSCs) is increasing worldwide. While NMSCs are more common, melanoma remains the most challenging because of its higher aggressiveness. Although the use of sunscreens is key in high-risk populations, it provides limited protection, which highlights the need for alternative solutions. In this review, we discuss current evidence on chemopreventive therapies, as well as their efficacy and adverse events, including immunocompetent and immunosuppressed patients. Acitretin, nicotinamide, 5-fluorouracil, and photodynamic therapy have shown overall promising results in actinic keratosis and squamous cell carcinoma. Nevertheless, more research is needed to establish their efficacy, particularly in melanoma, Merkel cell carcinoma, and cutaneous lymphoma, due to their higher mortality rates.

## Simple Summary

Chemopreventive agents in cutaneous oncology target the prevention of skin cancer and the active treatment of precancerous lesions. The ideal chemopreventive agent should be both safe and effective, have minimal interactions, and, to improve patient compliance, be easy to use and inexpensive. Although several drugs have been studied, the main obstacles for the assessment of their efficacy are difficulties in the design and conduction of clinical trials, including sample sizes and long follow-ups. To date, acitretin, nicotinamide, 5-fluorouracil, and photodynamic therapy have shown promising results and are currently used in clinical practice.

## 1. Introduction

Keratinocyte cancers or non-melanoma skin cancers (NMSC), including basal cell carcinoma (BCC) and skin squamous cell carcinoma (SCC), are those most frequently diagnosed and have shown increasing incidence over the past decades, representing significant costs for health care systems [1,2]. They are mostly caused by ultraviolet (UV) radiation, both by UVB through direct DNA damage and by UVA through oxidative stress and indirect DNA damage. BCC does not present a precursor lesion and usually follows a “benign course”, with a slow progression and rarely causes metastatic disease. However, SCC presents an in situ or precancerous state, referred to as actinic keratosis (AK), and has a poor prognosis in the metastatic state. While NMSC-associated mortality is lower compared to other malignancies, these cancers can provoke significant morbidity as they usually require surgery that may lead to disfigurement (when occurring in visible areas) and/or surgical complications. As for NMSCs, melanoma is mostly a UV-related tumor (approximately 95% of cases) and, although less common, it accounts for 90% of the deaths associated with cutaneous tumors [3]. Melanoma is at the center of most secondary prevention screening campaigns [4].

In addition to early detection strategies, and although less established, skin cancer prevention also encompasses chemoprevention. Chemoprevention can be defined as the “inhibition or reversal of pre-invasive carcinogenesis before cellular invasion across the basement membrane, using natural or pharmacologic agents” [5]. Therefore, chemopreventive agents target either the prevention of the incidence of future skin cancer (inhibition) or the active treatment of precancerous states, such as the treatment of AKs (reversal). The ideal chemopreventive agent should be both safe and effective, have minimal interactions, and, to improve patient compliance, be easy to use and inexpensive. Several drugs have been studied, but the main obstacles for the assessment of their efficacy comprise difficulties in the design and conduction of clinical trials, including sample sizes and long follow-ups. There is, therefore, a dearth of data on the chronic use of chemopreventive agents. Finally, most of the available drugs have been shown to lose their efficacy after treatment withdrawal [6]. It is generally agreed that the target populations for chemoprevention are patients with medium-to-high risk of developing skin malignancies, meaning patients with a history of skin cancer and/or immunosuppressed patients (including solid organ transplant recipients, SOTRs).

This narrative review critically evaluates the current state of knowledge on chemopreventive agents, identifying gaps and controversies and summarizing future directions. We conducted targeted literature searches in PubMed/MEDLINE and Google Scholar for English-language articles published up to 15 December 2025 using combination of the following keywords: “chemoprevention”, “skin cancer”, “non melanoma skin cancer”, “squamous cell carcinoma”, “actinic keratosis”, “basal cell carcinoma”, “melanoma”, “solid organ transplant recipient”, “retinoids”, “acitretin”, “isotretinoin”, “vitamin A”, “nicotinamide”, “capecitabine”, “arachidonic acid”, “difluoromethylornithine”, “5-fluorouracil”, “imiquimod”, “diclofenac sodium”, “ingenol mebutate”, “photodynamic therapy”, “tirbanibulin”, “tretinoin”, “vitamin D”, “polyphenols”, “T4 Endonuclease V”, “ablative fractional laser therapy”, “metformin”, “carvedilol”, “photoprotection”, “sunscreen”. Additional relevant studies were identified by screening the reference lists of key articles. Articles were included if they published evaluable data relevant to the scope of chemoprevention in skin cancer or addressed purely cosmetic or non-oncologic indications. Titles and abstracts were initially screened for relevance, followed by full-text review of selected articles. In addition, regulatory documents and safety communications from the European Medicines Agency and the U.S. Food and Drug Administration were reviewed. The selected articles were added to various sections as presented in our review. GenAI (GPT-5.2) and Biorender were used to assist in the generation of graphics.

## 2. Systemic Agents

Many systemic agents have been studied for the chemoprevention of skin cancer, including retinoids and vitamin A, nicotinamide, capecitabin, cyclooxygenase-2 inhibitors, and difluoromethylornithine.

### 2.1. Retinoids

#### 2.1.1. Acitretin

**Figure 1:**
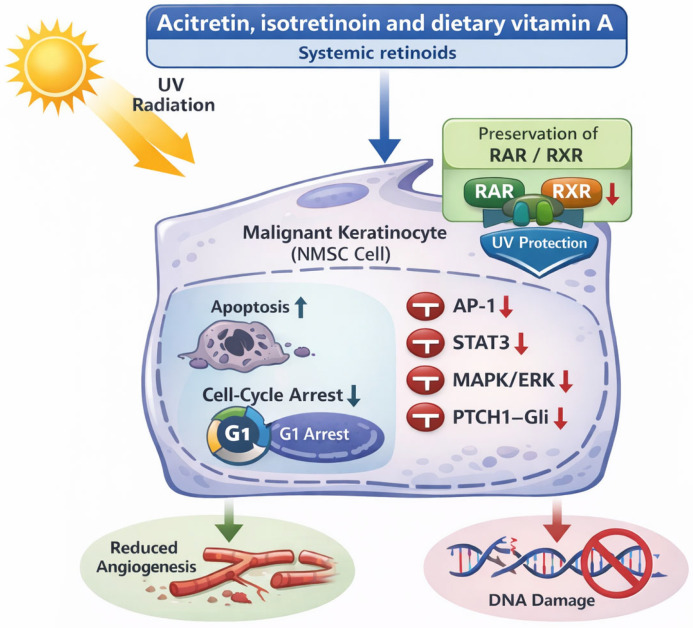
Mechanisms of action of retinoids in the prevention of non-melanoma skin cancer (NMSC). This figure illustrates the multimodal chemopreventive actions of systemic retinoids in NMSC, including direct antitumor effects on keratinocytes, inhibition of oncogenic signaling pathways, modulation of the tumor microenvironment, and protection against UV-mediated carcinogenesis.

Acitretin is a systemic retinoid that has been largely evaluated as a chemopreventive agent for NMSC, particularly among SOTRs and patients with chronic hematopoietic disorders [7,8]. Acitretin may prevent NMSC by inducing apoptosis and cell-cycle arrest in malignant keratinocytes while suppressing key pathways such as AP-1, STAT3, MAPK/ERK, and PTCH1–Gli. It also reduces angiogenesis and protects against UV-induced damage by preserving retinoid receptor expression (Figure 1) [9].

Randomized trials and systematic reviews have shown that acitretin substantially reduces keratinocyte carcinoma incidence in immunosuppressed individuals [7,10,11,12]. In a double-blind, placebo-controlled trial in renal transplant recipients, acitretin 30 mg/day for six months reduced the incidence of new SCCs to 11% versus 47% with placebo [10]. An open-label randomized crossover trial in 23 renal allograft recipients showed that one year of acitretin 25 mg/day significantly reduced SCC incidence compared to a preceding drug-free year [7]. A systematic review corroborated these findings, demonstrating a significant reduction in the incidence of new NMSC among renal transplant recipients (RR 0.22; 95% CI 0.06–0.90) [11]. Long-term cohort data also showed ≥50% reductions in keratinocyte carcinoma incidence over five years, with lower median dosing (10 mg/day) [12]. In SOTRs, low-dose regimens halved the incidence of new keratinocyte carcinomas, with reductions in both SCCs and BCCs [13]. Another cohort study showed 68% reduction in invasive SCCs in immunocompetent patients and SOTRs with high tumor burden [14]. Based on these findings, a Delphi consensus recommended acitretin as a chemopreventive agent for solid organ transplant recipients with high SCC burden or high-risk tumors [15]. In patients with chronic hematopoietic diseases treated with acitretin, recent analyses of large datasets similarly showed reduced NMSC rates [8].

Evidence in non-transplant high-risk populations is more limited. A randomized controlled trial in immunocompetent high-risk patients found that acitretin was associated with fewer new SCCs and BCCs than placebo; however, it did not significantly delay or reduce the cumulative incidence of NMSC [16].

The primary limitation of acitretin is its adverse-event profile, which includes mucocutaneous symptoms, hyperlipidemia, and liver function abnormalities [7,17]. Although most adverse events are mild, tolerability remains a challenge, particularly at higher doses [18]. Benefits may decrease after discontinuation, with tumor rates increasing after withdrawal [7]. To date, no standardized dosing or duration guidelines exist, underscoring the need for individualized risk-benefit assessment.

In conclusion, acitretin may be considered a chemopreventive agent, particularly in immunosuppressed patients. The optimal dose must be adjusted to balance efficacy against adverse events. Starting doses of 10 mg/day, with laboratory monitoring, and up to 25 mg/day, if well tolerated, may be indicated.

#### 2.1.2. Isotretinoin (13-Cis-Retinoic Acid)

Isotretinoin has not demonstrated efficacy for chemoprevention of NMSC in the general high-risk population. Large randomized, placebo-controlled trials using low-dose isotretinoin (5 mg–10 mg/day for three years) found no reduction in the incidence of either BCC or SCC and reported frequent mucocutaneous and metabolic adverse events [19,20].

In contrast, systemic retinoids may benefit selected high-risk groups, particularly SOTRs with multiple or recurrent NMSC, as supported by observational studies and expert reviews [21]. The chemopreventive effect seems to be limited to active treatment, with tumor recurrence often following discontinuation. Acitretin is preferred due to its pharmacokinetic profile, but isotretinoin is used off-label when acitretin is contraindicated or unavailable. Optimal dosing is undefined, although low-dose initiation with titration based on tolerance is recommended. Long-term therapy is constrained by toxicity.

In conclusion, the use of systemic isotretinoin in chemoprevention remains limited to transplant recipients, with current practice guided primarily by expert consensus rather than randomized controlled trials.

#### 2.1.3. Dietary Vitamin A

High dietary vitamin A intake was associated with a modest reduction in cutaneous SCC risk in a study on self-reported data on medical history and lifestyle [22]. Trials of oral retinol (25,000 IU/day) demonstrated a reduced occurrence of the first SCC in individuals with moderate risk, but no benefit for BCC or high-risk groups, and adverse events limit its broader use [23]. No protective effect has been observed for melanoma.

In conclusion, current guidelines do not recommend vitamin A supplementation. Moreover, high preformed vitamin A intake may increase the risk of osteoporosis [24].

### 2.2. Nicotinamide

**Figure 2:**
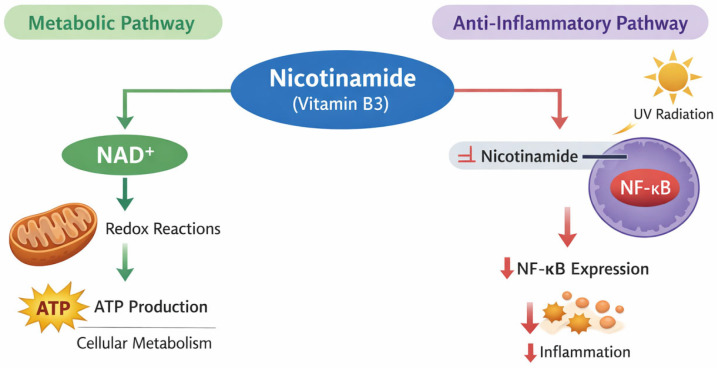
Mechanisms of action of nicotinamide. Nicotinamide functions as a precursor of nicotinamide adenine dinucleotide (NAD^+^), thereby supporting redox reactions essential for ATP production and cellular metabolic processes. Additionally, nicotinamide modulates inflammatory signaling by decreasing NF-κB expression after ultraviolet (UV) radiation exposure, resulting in reduced inflammation.

Nicotinamide is an amide form of vitamin B3 and the precursor of nicotinamide adenine dinucleotide (NAD^+^), an essential co-enzyme of redox reactions for adenosine triphosphate (ATP) production and for other metabolic processes [25]. Nicotinamide also has an anti-inflammatory role by decreasing the expression of NF-kB after UV radiation (Figure 2). In animal models, NAD^+^ deficiency led to UV sensitivity of the skin, impaired DNA damage response, increased genomic instability, and cancer incidence. Moreover, NAD^+^ is depleted with age and/or following UV radiation [25,26]. Therefore, nicotinamide supplementation should increase DNA repair and reduce UV immunosuppression with few adverse events [27,28].

Nicotinamide is one of the most studied drugs for the chemoprevention of skin cancer. Randomized trials have shown a reduction in the rate of AKs and NMSC in high-risk patients [29,30]. However, these results have not always been significant. Interestingly, a case–control study conducted by Drago et al. suggested that nicotinamide could also be used as a treatment for AKs [31].

The ONTRAC trial was a phase III clinical study comparing the use of nicotinamide 500 mg twice daily (n = 192) vs. placebo (n = 191) during a period of 12 months [30]. The target population was high-risk patients who had had at least two NMSCs in the previous 5 years. The primary endpoint was the number of new NMSCs. Results showed that the rate of new NMSCs and AKs was 23% lower in the nicotinamide group than in the placebo group. No significant differences were observed for BCC. Of note, there was no significant decrease in recurrence rates at 6 months after treatment discontinuation, suggesting that nicotinamide is effective only during treatment, without long-term effects after withdrawal. The investigators observed a trend toward greater effectiveness of nicotinamide among patients who had had a higher number of NMSCs in the past 5 years. Regarding toxicities, more mucocutaneous infections were noticed in the nicotinamide group [30,32].

The ONTRANS trial compared the use of nicotinamide 500 mg 2/d (n = 79) vs. placebo (n = 78) for 12 months in SOTRs who had had at least two NMSCs in the previous 5 years [33]. The primary endpoint was the number of new NMSCs. Unfortunately, the study was underpowered since it was terminated prematurely due to poor recruitment, and the results showed no differences between the groups.

Based on these results, the European consensus-based interdisciplinary guideline for invasive cutaneous SCC stated that nicotinamide 500 mg twice daily may be offered to immunocompetent patients with a history of multiple cutaneous SCCs, considering the favorable safety profile (grade of recommendation C, level of evidence 3) [34].

Recently, Breglio et al. conducted a large retrospective study to determine the efficacy of nicotinamide for skin cancer prevention, including SOTR [35]. A total of 12,287 patients were exposed to oral nicotinamide 500 mg twice daily for longer than 30 days. Overall, there was a significant 14% reduction in BCC and SCC, with the greatest risk reduction seen for SCC. When nicotinamide was initiated after the first skin cancer, the risk reduction was 54%, with a decline following subsequent skin cancers. No overall significant risk reduction was observed in SOTR, although early nicotinamide use was associated with reduced SCC incidence.

Hwang et al. performed a retrospective cohort study on 47 SOTRs to assess the effectiveness of nicotinamide 500 mg twice daily, for at least one year, in the secondary prevention of NMSC [36]. The primary outcome was NMSC incidence in the year before and after nicotinamide supplementation. Secondary outcomes included the incidence of SCC and BCC at one- and two-year intervals. The investigators found a mean reduction of 2.2 NMSCs at one and two years post-nicotinamide supplementation. Of note, significant reductions were observed in SCC, but a decrease in BCC was not statistically significant. Therefore, the authors recommended nicotinamide as a low-risk and low-cost chemopreventive agent for reducing SCC in SOTRs.

Finally, there is an ongoing clinical trial (NCT05955924) on the use of nicotinamide as chemoprevention for NMSC in SOTR. It is a multicenter study involving transplant centers in Canada to evaluate nicotinamide 500 mg twice daily vs. placebo for up to 4 years in 396 patients. The primary endpoint is the reduction rate of further NMSC. Study completion is expected in August 2027. A summary of the main studies conducted on nicotinamide is shown in Table 1.

In conclusion, nicotinamide has demonstrated its efficacy as a chemopreventive agent in NMSC, both in immunocompetent and immunocompromised patients, and it is currently used in clinical practice. As with retinoids, nicotinamide’s protective effects disappear after treatment cessation; it has been postulated, therefore, that nicotinamide might significantly slow the process of cancerization in already affected cells. The results of clinical trial NCT05955924 will provide stronger and long-term data in SORT, including BCC. As highlighted with other chemopreventive agents, clinical trials on the efficacy of nicotinamide in melanoma incidence are lacking.

The reported adverse effects of nicotinamide are mostly mucocutaneous infections. Contrary to retinoids, no laboratory monitoring is required. Of note, an interaction may exist between nicotinamide and carbamazepine, probably due to the inhibition of cytochrome p-450 by nicotinamide [37]. Therefore, this combination should be avoided.

### 2.3. Capecitabine

**Figure 3:**
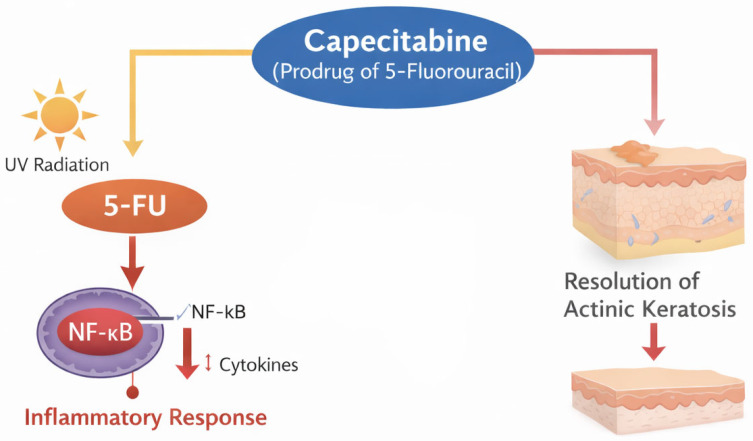
Mechanisms of action of capecitabine. Capecitabine, an oral prodrug of 5-fluorouracil, can induce inflammation and promote the resolution of actinic keratosis following ultraviolet (UV) radiation exposure.

Capecitabine is an oral prodrug of 5-fluorouracil. It has been seen to induce inflammation and even resolution of AKs in patients treated for colon and breast cancer (Figure 3) [38,39]. It may also be associated with a decrease in AK, BCC, and SCC incidence [39]. However, evidence is limited to small series, and no optimal dosing has been established. Toxicities include fatigue, nausea, vomiting, diarrhea, high creatinine levels, hand–foot syndrome, hyperuricemia, weight loss, anemia, and cardiomyopathy [39].

Regarding SOTRs, retrospective studies have been conducted to determine the efficacy of low-dose capecitabine (0.5–1.5 g/m^2^ per day) in the secondary prevention of skin cancer in this population [40,41]. SOTRs experienced a statistically significant decline in SCC, BCC, and AK incidence during treatment [41]. The most common grade 3 and 4 toxicities included fatigue (40.0%), hand–foot syndrome (20.0%), and diarrhea (20.0%). The discontinuation rate at one year was 33.3% [41].

In conclusion, capecitabine could be considered as chemoprevention in SOTRs. Health care professionals must weigh the benefits against the risk of toxicities for each patient individually. Further investigation, including prospective clinical trials, is needed.

### 2.4. Compounds That Inhibit the Arachidonic Acid Pathway

**Figure 4:**
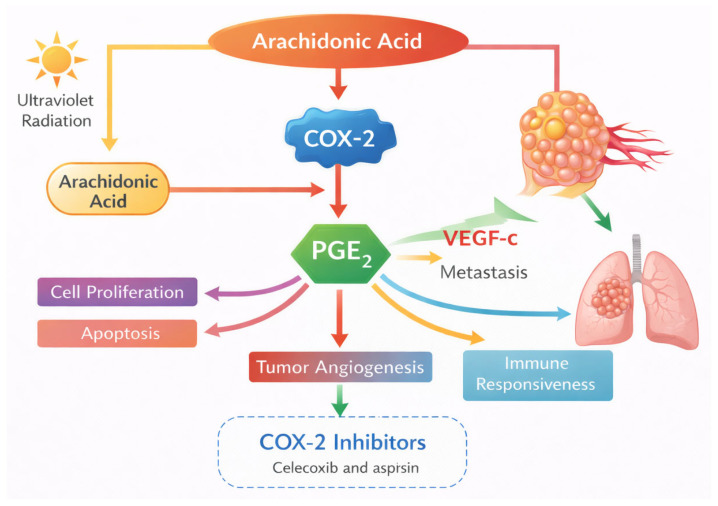
Mechanisms of action of COX-2 inhibitors in the arachidonic acid pathway. Ultraviolet (UV) radiation induces arachidonic acid metabolism and upregulation of cyclooxygenase-2 (COX-2), leading to increased levels of prostaglandin E_2_ (PGE_2_). PGE_2_ promotes cell proliferation, apoptosis, tumor angiogenesis, immune responsiveness, and metastasis via VEGF-c. COX-2 inhibitors, such as celecoxib and aspirin, block this pathway, thereby modulating these processes and potentially reducing cancer progression.

Ultraviolet radiation on the skin induces prostaglandin production and cyclooxygenase-2 (COX-2) expression. COX-2 is associated with tumor initiation and progression in many cancer types [42]. COX-2 expression and its derived prostaglandin 2 (PGE2) levels are correlated with cancer development and metastases. More precisely, PGE2 is involved in cell proliferation, apoptosis, tumor angiogenesis, and immune responsiveness. COX-2 is associated with VEGF-c overexpression, contributing to metastasis in various types of cancers (Figure 4) [42].

Therefore, nonsteroidal anti-inflammatory drugs (NSAIDs) have been studied in chemoprevention. Aspirin has been demonstrated to be chemopreventive in colon cancer, and it has also shown promising results in skin cancer in mouse models [43]. However, conflicting data have been published regarding its use as chemoprevention of skin cancer in humans.

Tang et al. found that the COX-2 inhibitor celecoxib decreased the development of new BCCs in patients with PTCH1 +/− basal cell nevus syndrome, but it did not reach statistical significance [44].

Elmets et al. conducted a randomized controlled trial on celecoxib (200 mg 2/d, n = 87) vs. placebo (n = 96) [45]. It was a 9-month intervention on patients who had 10 to 40 AKs. The primary endpoint was the number of new AKs. However, the study was terminated prematurely due to Food and Drug Administration (FDA) concerns about cardiovascular risks. Results found no difference in the incidence of AKs at 9 months. However, at 11 months after randomization, there were fewer NMSCs in the celecoxib arm than in the placebo arm, and cardiovascular toxicities were similar in the two groups. Hence, celecoxib may be effective for the prevention of SCCs and BCCs in patients who have major actinic damage and are at risk of developing NMSCs.

Conversely, a meta-analysis conducted by Zhang et al. showed no chemopreventive effect, either from aspirin or from COX-2 inhibitors, on NMSC, and their use is currently not recommended [46]. Moreover, as stated, NSAIDs carry an increased risk of cardiovascular toxicities, including renal insufficiency, hypertension, gastrointestinal symptoms, bleeding, and thromboembolic events that limit their chronic use.

Of note, there was significant heterogeneity in the publications analyzed, and most were observational studies, which were unable to control for important potential confounders. Muranushi et al. led a systematic review and found that the use of non-aspirin NSAIDs reduced the risk of developing SCC by 18% [47]. A reduced risk of similar magnitude was also observed among aspirin users, although with borderline statistical significance. The authors found significant heterogeneity among studies regarding SCC risk estimates resulting from aspirin use and any NSAID use.

Regarding melanoma, in a randomized trial, Yan et al. concluded that aspirin was not associated with a reduced risk of invasive melanoma in older individuals [48]. Additional studies are required to explore this relationship further.

Finally, Ikeya et al. suggested that in patients taking voriconazole, chemoprevention with selective COX-2 inhibitors may be helpful to repress the development of skin cancers derived from DNA-damaged keratinocytes [49]. Indeed, voriconazole induces the development of UV-associated skin cancers, stimulating aryl hydrocarbon receptors and upregulating COX-2.

In conclusion, evidence on the use of NSAIDs as a chemopreventive agent in skin cancer is inconsistent. The summarized findings suggest that inhibition of the arachidonic acid pathway has the potential to prevent the development of cutaneous SCC, but this is probably limited to selected patients. Group stratification might be needed to find conclusive results. Furthermore, data on melanoma are still lacking, and the risk of cardiovascular toxicities should also be taken into consideration.

### 2.5. Difluoromethylornithine

**Figure 5:**
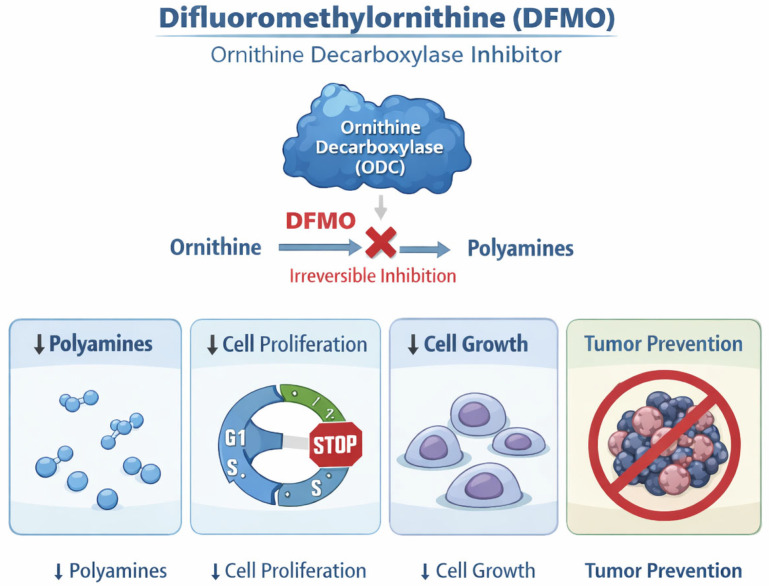
Mechanism of action of difluoromethylornithine (DFMO) in cancer prevention. Difluoromethylornithine (DFMO) acts as an irreversible inhibitor of ornithine decarboxylase (ODC), a key enzyme in polyamine biosynthesis. Inhibition of ODC leads to reduced intracellular polyamine levels, resulting in decreased cell growth and proliferation. Evidence from preclinical models suggests that suppression of polyamine synthesis by DFMO limits tumor initiation and prevents tumor formation.

Difluoromethylornithine (DFMO) is an irreversible inhibitor of the ornithine decarboxylase enzyme involved in polyamine synthesis, needed for cell growth and proliferation (Figure 5) [50]. Evidence coming from preclinical models suggests that inhibition of the ornithine decarboxylase will prevent tumor formation [50]. Several studies have focused on the use of DMFO as a chemopreventive agent in a range of cancers, including skin cancers, with variable results. The longest randomized trial comparing systemic DFMO to placebo in patients with a prior history of NMSC did not meet its primary endpoint of reducing new NMSC [51,52]. Moreover, in the long term, DFMO has been shown to be ototoxic, causing reversible hearing loss [53]. No new studies on its use are currently ongoing. At present, its use is not recommended for skin cancer prevention/treatment [54].

## 3. Topical and Field Therapies

Topical and field therapies are considered secondary prevention strategies as they are used mainly as treatments for AKs. They are used as intermittent short-course treatments and not as chronic treatments. Here we include 5-fluorouracil, imiquimod, diclofenac sodium, ingenol mebutate, photodynamic therapy, and the recently introduced tirbanibulin.

### 3.1. 5-Fluorouracil

**Figure 6:**
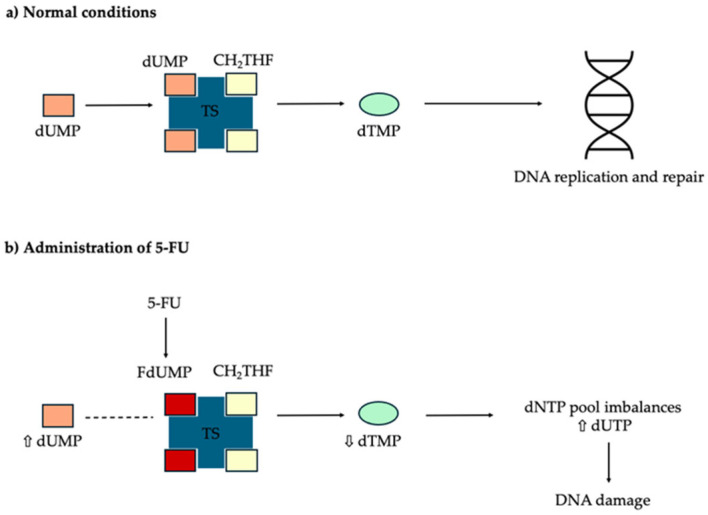
Mechanism of action of 5-fluorouracil (5-FU). Thymidylate synthase (TS) facilitates the conversion of deoxyuridine monophosphate (dUMP) into deoxythymidine monophosphate (dTMP), using 5,10-methylene tetrahydro-folate (CH2THF) as the methyl group donor. The active metabolite of 5-fluorouracil (5-FU), fluorodeoxyuridine monophosphate (FdUMP), binds to TS at its nucleotide-binding site and forms a stable ternary complex with TS and CH2THF. This complex prevents dUMP from accessing the binding site, thereby inhibiting dTMP production. As a result, the balance of deoxynucleotide triphosphates (dNTP) is disrupted, and deoxyuridine triphosphate (dUTP) levels rise, both of which lead to DNA damage.

5-fluorouracil (5-FU) is a pyrimidine analog that inhibits thymidylate synthase, thereby interfering with DNA and RNA synthesis (Figure 6) [55,56]. It preferentially targets dysplastic keratinocytes with high proliferative activity. Substantial evidence supports the ability of 5-FU to reduce AK burden [57].

As there is a well-established progression sequence from AK to SCC [58], much of the existing literature uses AK reduction as a surrogate marker for decreased SCC risk. However, data directly demonstrating prevention of invasive NMSC remain relatively scarce and long-term studies with cancer incidence as the primary endpoint are needed. 5-FU is therefore considered one of the main treatments for AK and serves not only as a lesion-directed therapy but also as a field-directed chemopreventive option for NMSC. Available formulations include 5% 5-FU cream, 4% 5-FU in aqueous cream, 0.5% 5-FU in salicylic acid 10% solution, and 5% 5-FU plus calcipotriol 0.005% cream [57].

Pomerantz et al. evaluated the long-term efficacy of a single course of 5% 5-FU cream for AK treatment in the Veterans Affairs Keratinocyte Carcinoma Chemoprevention (VAKCC) trial [59]. Patients were randomized to apply either topical 5-FU or the vehicle control cream to the face and ears twice a day for up to 4 weeks. A significant and sustained reduction in AK burden after a single treatment cycle was observed, with benefits maintained for more than two years, including a reduced incidence of new AK over time. In clinical practice, 5% 5-FU cream is topically applied once or twice daily for 3–4 weeks when treating AK [59].

Other studies have similarly supported the efficacy of lower-strength formulations. Topical 4% 5-FU in aqueous cream applied once daily for 4 weeks achieved complete and ≥75% lesion clearance rates, comparable to 5% 5-FU cream applied twice daily, with fewer severe local skin reactions [60]. This formulation is generally applied once daily on grade I-II AK located on the face, ears, and scalp. Two randomized trials by Jorizzo et al. and Weiss et al. showed that 0.5% 5-FU cream applied for one, two, or four weeks significantly reduced lesion counts compared with placebo [61,62].

A combination of 0.5% 5-FU with 10% salicylic acid showed superior efficacy for hyperkeratotic AK in a Phase III randomized, double-blind, vehicle-controlled trial [63], probably because the keratolytic effect of salicylic acid enhances drug penetration. This solution is applied once daily until lesion clearance or for up to 12 weeks [63].

Another emerging strategy is the combination of 0.005% calcipotriol ointment plus 5% 5-FU cream for field-directed treatment of AK. In a recent systematic review, this regimen achieved a greater AK clearance in treated areas compared to 5-FU plus petroleum jelly [64]. Over a 3-year follow-up, the combination was associated with a lower risk of SCC on the face and scalp. Although these findings are promising, evidence directly linking this regimen to reduced SCC incidence remains limited and requires further prospective studies.

The safety profile of topical 5-FU is well characterized. Local skin reactions are nearly universal but typically self-limiting. Pain, erythema, scaling, crusting, and erosions are common, but systemic toxicity is exceptional. The intensity of local skin reactions induced by 5-FU increases proportionally with the number of AK lesions at baseline [65]. This reinforces the concept that the visible inflammatory response, inherent to the elimination of atypical keratinocytes, reflects pharmacologic activity and may serve as a clinical marker of treatment effectiveness.

In conclusion, topical 5-FU remains a practical, low-cost, and effective treatment of AKs. We could hypothesize that, through its ability to reduce AK burden and treat field cancerization, it could further serve as a chemopreventive agent for skin cancer. New formulations and combination regimens—particularly with salicylic acid and calcipotriol—are broadening its therapeutic potential, and shorter courses may still confer preventive benefit, an important consideration for improving adherence in patients requiring repeated field treatments over long periods.

Although stronger prospective evidence is still needed to directly quantify its impact on SCC incidence, current data support its use in high-risk patients with extensive actinic damage, who may require repeated treatment cycles. Clinicians should individualize treatment plans based on patient risk, lesion burden, tolerability, and preference to optimize preventive outcomes.

### 3.2. Imiquimod

**Figure 7:**
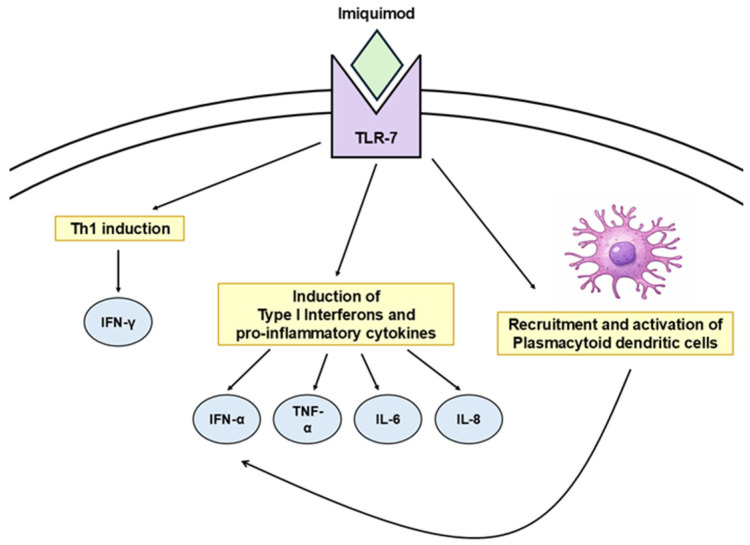
Mechanism of action of topical imiquimod. Imiquimod activates toll-like receptor 7, inducing a Th1-type immune response, the production of type I interferons and pro-inflammatory cytokines, and the recruitment of plasmacytoid dendritic cells, which contribute to amplification of local immune responses. TLR7, toll-like receptor 7; IFN, interferon; TNF, tumor necrosis factor; IL, interleukin; Th1, T helper type 1.

Imiquimod is a toll-like receptor-7 agonist that acts as an immune response modifier with both antiviral and antitumoral activity [66]. It activates innate immunity, characterized by a strong induction of type I interferons—especially IFN-α—together with pro-inflammatory cytokines such as TNF-α, IL-6, and IL-8 [67]. A central mechanism involves the recruitment and activation of plasmacytoid dendritic cells, the main source of cutaneous type I interferons, which amplifies local immune activity and promotes the clearance of dysplastic or virally altered cells [68]. In addition, imiquimod can enhance adaptive immunity through increased Th1-related cytokine production (Figure 7).

As with other topical therapies, imiquimod acts through the treatment of AK and UV-damaged skin within the cancerization field, effectively reducing both clinical and subclinical dysplasia. However, whether this translates into a lower incidence of invasive SCC remains uncertain.

Imiquimod is available in 5% and 3.75% formulations. The 5% cream is applied three times per week in treatment courses of 4–8 weeks—although extended 12–16-week regimens are also used in clinical practice—and is approved for areas of up to approximately 25 cm^2^ on the face and scalp [57,69]. In contrast, the 3.75% formulation can be used on larger fields of up to 200 cm^2,^ and it is applied once daily in two 2-week cycles separated by a 2-week rest interval [57].

Across randomized and observational studies, imiquimod demonstrated consistent efficacy in reducing AK burden. In a recent systematic review, overall AK reduction after treatment with imiquimod averaged 67.5% ± 19.6 at 1–3 months, 64.0% ± 13.0 at 3–6 months, and 68.0% ± 1.6 at 6–12 months [70]. In a separate network meta-analysis, treatment with imiquimod for 16 weeks yielded an estimated complete clearance rate of 63.3% (95% CI: 45.5–81.1), compared with 56.3% (95% CI: 33.8–78.8) for a 4-week regimen and approximately 36% for the 3.75% formulation, indicating higher efficacy with longer 5% courses [71]. Recurrence rates at 12 months among patients who achieved complete clearance at 2–3 months ranged between 27% and 39% [72].

Adverse events of imiquimod mainly include local inflammatory reactions such as erythema, pain, erosion, crusting, and pruritus, which typically resolve after treatment cessation and reflect dermal immune activation. Systemic flu-like symptoms—including fever, fatigue, myalgia, and headache—may occur, particularly with the 5% formulation, but are infrequent [73]. Imiquimod may also trigger or exacerbate autoimmune or inflammatory dermatoses, such as vitiligo, psoriasis, erythema multiforme, or lichen planus, warranting caution in patients with personal or family history of these diseases [74]. There has been concern around the immune stimulation effect of imiquimod in SOTRs; however, evidence suggests that its use is safe in this population [75,76,77,78]. In general, treatment with imiquimod is not recommended during the summer period [79], but it is well tolerated overall, and discontinuation due to adverse effects is uncommon [73].

In conclusion, even though imiquimod has not been shown to reduce the incidence of invasive SCC, it is an effective field-directed therapy capable of reducing both visible AK and subclinical dysplasia within photodamaged skin. However, its efficacy is consistently lower than that of 5-FU, which shows higher and more sustained clearance in comparative studies [80]. Indirect comparisons also suggest that imiquimod performs better than diclofenac formulations and achieves outcomes broadly comparable to photodynamic therapy (both later discussed), depending on regimen and treatment duration [70,71]. Within its formulations, the 5% regimen achieves higher clearance than the 3.75% formulation, particularly when used in longer treatment courses. Although the 5% cream is limited by the maximum treatable surface area, sequential treatment of adjacent zones can bypass the surface area limitations.

### 3.3. Diclofenac Sodium 3%

**Figure 8:**
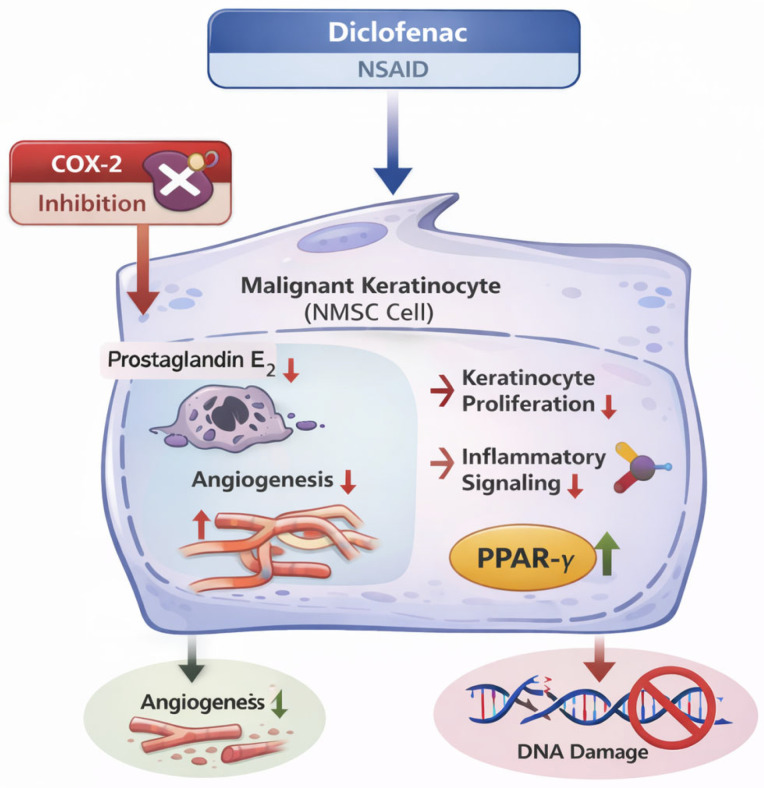
Mechanisms of action of diclofenac in the chemoprevention of non-melanoma skin cancer (NMSC). Diclofenac, a nonsteroidal anti-inflammatory drug (NSAID), exerts chemopreventive effects in NMSC, primarily through selective cyclooxygenase-2 (COX-2) inhibition. This leads to reduced prostaglandin E_2_–mediated angiogenesis, keratinocyte proliferation, and pro-tumorigenic inflammatory signaling involved in early stages of cutaneous carcinogenesis. Additional differentiation-modulating effects, including partial activation of peroxisome proliferator-activated receptor gamma (PPAR-γ), may further contribute to its preventive activity.

Diclofenac is an NSAID whose antitumoral activity is mainly mediated through selective COX-2 inhibition, reducing prostaglandin E_2_–driven angiogenesis, keratinocyte proliferation, and inflammatory signaling implicated in early cutaneous carcinogenesis [81]. Additional differentiation-modulating mechanisms, including partial PPAR-γ activation, have also been proposed (Figure 8) [81].

Diclofenac has well-established efficacy for the field treatment of AK. Histologic studies have demonstrated that diclofenac induces significant regression of keratinocytic atypia, including lowering of AK grade, marked reduction in mitotic figures, and decreased expression of p53 and Ki-67, indicating partial reversal of early neoplastic changes within actinically damaged skin [82]. Importantly, complete histologic clearance has been observed in approximately 23% of treated lesions after a 90-day course, even when complete clinical clearance is not achieved [82].

More recent real-world data have extended these findings. In a multicenter longitudinal cohort of 172 patients with multiple AKs, diclofenac achieved 18–29% complete clinical clearance at 12 months, with progressive improvement maintained beyond treatment completion, consistent with a slow but sustained remodeling of the cancerization field [83]. Tolerability was excellent, with only mild local skin reactions, reinforcing its suitability for large or cosmetically sensitive areas and for repeated courses [83].

Evidence from high-risk populations further supports its secondary chemopreventive potential. In a randomized, placebo-controlled study in SOTRs, diclofenac achieved significantly higher AK clearance versus vehicle (41% vs. 0%), and, notably, no invasive SCCs developed within treated fields over 24 months, despite frequent AK recurrence [84]. Although SCC incidence was not a predefined endpoint, the absence of progression in a severely immunosuppressed cohort supports a role for diclofenac in preventing malignant transformation within high-risk actinic fields.

Contemporary evidence confirms that while diclofenac effectively targets dysplastic keratinocytes and improves field cancerization, no robust clinical trials have evaluated primary prevention of NMSC, as no studies have measured SCC/BCC incidence as a primary endpoint [81,85].

In conclusion, diclofenac should be considered a secondary chemopreventive agent (field treatment), capable of reducing progression risk within areas of established actinic damage.

### 3.4. Ingenol Mebutate

Ingenol mebutate (IM) is a topical diterpene ester derived from Euphorbia peplus that was approved for the treatment of AK and later withdrawn from the European market because of safety concerns [86]. It was approved by the FDA in 2012 and by the EMA in 2013 and was characterized by very short treatment regimens (2 or 3 days, depending on the anatomical location) and high efficacy in AK clearance.

**Figure 9:**
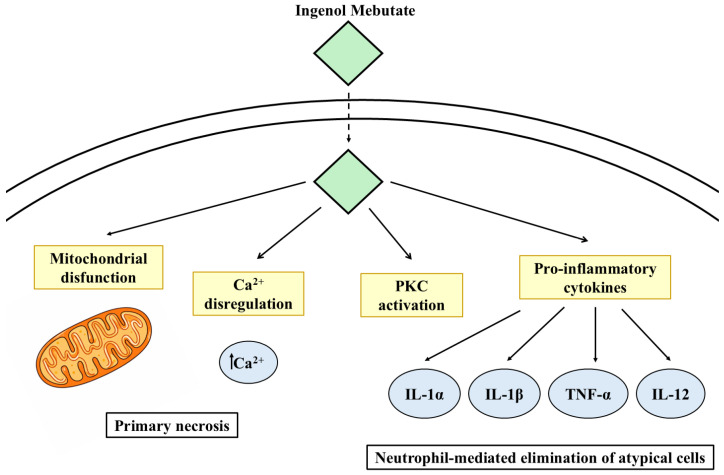
Mechanism of action of ingenol mebutate. Ingenol mebutate exerts a dual antitumor effect through a rapid direct cytotoxic mechanism mediated by plasma membrane disruption and mitochondrial dysfunction, leading to primary necrosis, and a secondary immune-mediated response characterized by protein kinase C activation, induction of pro-inflammatory cytokines, and neutrophil-mediated elimination of residual atypical cells. PKC, protein kinase C; IL, interleukin; TNF, tumor necrosis factor.

IM exerts its activity through a dual mechanism: a rapid direct cytotoxic effect on dysplastic keratinocytes, mediated by disruption of plasma membrane integrity and mitochondrial dysfunction, and a secondary immune-mediated effect driven by protein kinase C activation, with release of pro-inflammatory cytokines and recruitment of neutrophils that eliminate residual atypical cells (Figure 9) [86].

Phase III clinical trials demonstrated significantly higher clearance rates of AK compared with vehicle, supporting its initial approval as an effective field therapy [87]. However, a 3-year safety study showed a higher incidence of skin malignancies in IM-treated areas compared with imiquimod (3.3% vs. 0.4%), including an increased risk of invasive squamous cell carcinoma [88]. Based on these data, the EMA suspended its marketing authorization in 2020, concluding that its benefit–risk balance was no longer favorable [88]. Patients previously treated with IM should be monitored for the appearance of skin tumors in previous treatment fields.

### 3.5. Photodynamic Therapy

**Figure 10:**
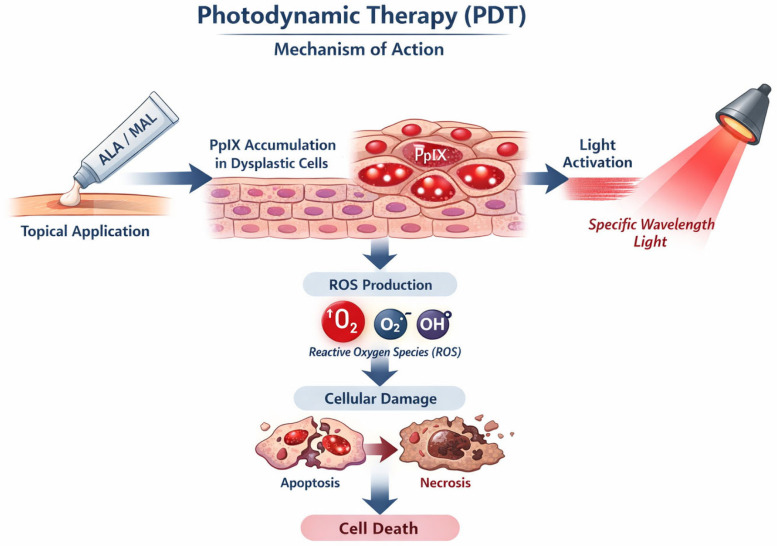
Mechanism of action of photodynamic therapy (PDT). Topically applied ALA or MAL is converted into protoporphyrin IX (PpIX), which accumulates in dysplastic keratinocytes. Light activation of PpIX induces the production of reactive oxygen species, causing oxidative damage and triggering apoptosis or necrosis, resulting in selective cell death.

Photodynamic therapy (PDT) is a minimally invasive treatment that combines the topical application of a photosensitizing agent with light exposure to generate reactive oxygen species (ROS) capable of selectively destroying target cells. The topical photosensitizers, most commonly used in dermatology, are 5-aminolevulinic acid (ALA) and its methyl ester derivative methyl aminolevulinate (MAL), which are precursors of the endogenous porphyrin protoporphyrin IX (PpIX). After topical application and incubation, PpIX preferentially accumulates within dysplastic or neoplastic keratinocytes. Upon illumination with a specific wavelength corresponding to its absorption spectrum, the photosensitizer triggers a photochemical reaction, leading to the production of reactive oxygen species and singlet oxygen, which are responsible for oxidative damage and subsequent apoptosis or necrosis of atypical keratinocytes (Figure 10) [89]. Although PpIX has a better absorption coefficient in the blue region of the spectrum, red light is usually preferred, particularly for thicker lesions, because of its greater depth of penetration in the skin [90]. In dermatology, PDT is employed for the management of AK and selected cases of NMSC, including superficial BCC and SCC in situ. However, the specific therapeutic indications vary depending on the photosensitizer and the regulatory framework of each country [89,91].

There is growing evidence that broad-field PDT may not only treat visible keratinocytic lesions but also prevent the development of new AK and NMSC. The biological effects of PDT extend beyond its direct cytotoxicity on dysplastic or malignant keratinocytes. The photosensitizer accumulates within vascular endothelial cells, leading to photochemical destruction of tumor-feeding microvessels, which has been shown to play a role in the long-term efficacy of PDT. Moreover, especially when applied with low light fluencies, PDT can enhance innate and adaptive immune responses through the release of pro-inflammatory mediators and subsequent recruitment and activation of neutrophils, macrophages, and dendritic cells [92]. In this regard, PDT has been shown to reduce epidermal dysplasia and solar elastosis while decreasing the expression of p53 and Ki67, markers of early carcinogenesis and cellular proliferation, respectively [93,94].

In hairless mice chronically exposed to solar-simulated UV radiation, MAL-PDT has demonstrated the ability to delay photocarcinogenesis compared with untreated controls [95]. Several clinical trials also support the preventive role of field-directed PDT in high-risk immunocompetent patients. In photodamaged patients with facial AKs and a previous history of NMSC, ALA-PDT could delay the appearance of new AKs by approximately 6 months compared with untreated areas [96]. In a larger randomized controlled trial (n = 166), after 52 weeks of follow-up, 3 sessions of ALA-PDT could decrease the number of AKs and NMSC compared with the vehicle arm (5 new NMSCs in 5/56 patients vs. 12 new NMSCs in 7/53 patients; *p* = 0.0014) [97].

SOTRs present an increased risk of NMSC, particularly SCC and AK. SOTRs often develop extensive cancerization fields on chronically photodamaged skin, which can be targeted with PDT. According to expert consensus statements and the 2019 European Dermatology Forum guidelines on PDT, the procedure may reduce the development of new AKs and the progression of existing lesions to invasive SCC in SOTR patients (Strength of recommendation B, Quality of Evidence I) [94]. In comparative studies, PDT has been shown to be comparable or superior to other field therapies, with complete clearance rates with MAL-PDT ranging from 40% to 76% [98]. However, the therapeutic response in SOTRs tends to be lower than in immunocompetent patients, probably due to impaired immune-mediated tumor clearance. For example, a randomized clinical trial reported a complete response rate at 48 weeks of 55% in SOTRs, compared with 72% in immunocompetent controls following conventional ALA-PDT [99]. Several clinical trials have assessed PDT as a preventive strategy in SOTRs, showing overall favorable, though varying, results [99,100,101,102,103,104]. A systematic review and meta-analysis obtained a pooled risk difference of 0.51 (95% CI 0.46–0.59), supporting its significant chemopreventive potential in SOTRs despite heterogeneity across trials [105].

Daylight photodynamic therapy (DL-PDT) is an alternative modality that requires natural sunlight instead of artificial monochromatic light for photosensitizer activation. This approach offers several advantages, including reduced treatment-related pain and the possibility of self-administration. This modality has been tested among SOTRs patients; in a split-face trial, six sessions of DL-PDT significantly reduced new lesion counts compared with cryotherapy [106].

In conclusion, despite the growing evidence supporting the preventive role of PDT in NMSC, most available studies suffer from important methodological limitations: heterogeneity of treatment protocols, small sample sizes, and short follow-up durations, which limit the generalizability of current findings. Economic considerations also remain relevant, as PDT requires specific equipment, trained personnel, and often multiple sessions. Long-term prevention of keratinocyte carcinoma will likely depend on treatment adherence, and thus, minimizing discomfort and pain may be as important as optimizing short-term efficacy. Future research is focusing on next-generation photosensitizers with improved quantum yield, deeper skin penetration, and enhanced safety profiles, as well as on novel delivery techniques such as laser-assisted drug administration to improve cutaneous uptake. Combining PDT with other topical, systemic, or physical modalities may increase its therapeutic and preventive potential. Larger, multicenter, randomized trials with standardized parameters and long-term follow-up, and the exploration of new photosensitizers and light sources will be key to defining the position of PDT within the landscape of skin cancer chemoprevention.

### 3.6. Tirbanibulin 1%

**Figure 11:**
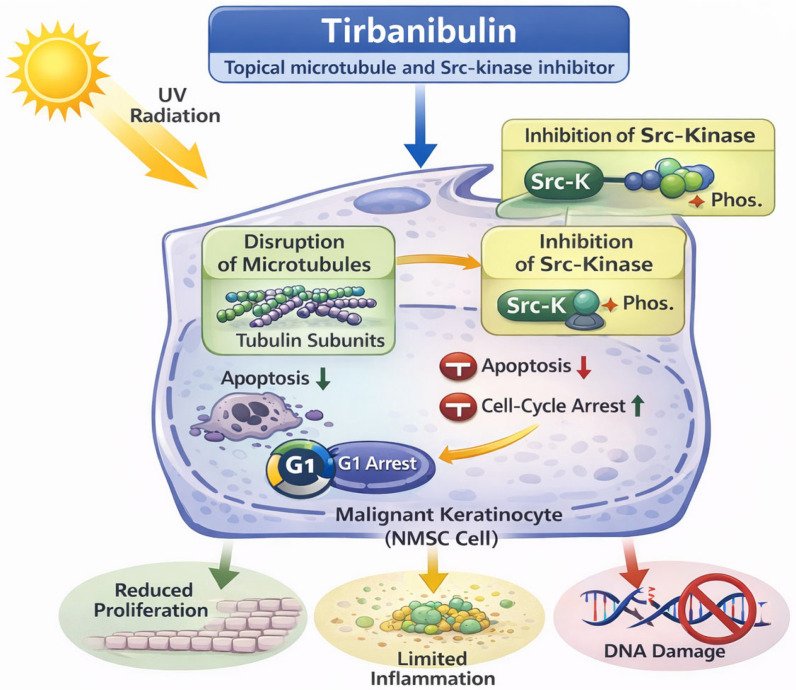
Mechanisms of action of tirbanibulin in the prevention of non-melanoma skin cancer (NMSC). Tirbanibulin inhibits microtubule polymerization and Src kinase-dependent signaling in atypical keratinocytes within actinically damaged skin. Disruption of microtubule dynamics induces mitotic arrest and apoptosis, while Src kinase inhibition suppresses downstream pathways involved in keratinocyte proliferation and survival. These combined effects result in selective elimination of dysplastic keratinocyte clones with a limited inflammatory response, contributing to control of field cancerization and reduction in NMSC risk.

Tirbanibulin 1% is a first-in-class topical microtubule and Src-kinase inhibitor approved for the field treatment of non-hyperkeratotic AKs on the face and scalp. By inducing rapid apoptosis of atypical keratinocytes with limited inflammatory response, tirbanibulin offers a short-course and highly tolerable option for patients requiring effective field therapy (Figure 11) [107,108].

The ointment is applied once daily for 5 consecutive days to a treatment area of up to 25 cm^2^, providing one of the shortest treatment durations among topical agents for AK.

Two identically designed Phase 3 randomized controlled trials, including 702 adults, demonstrated that tirbanibulin was significantly more effective than vehicle, achieving complete clearance in 44–54% of patients and partial clearance in 68–76% by day 57 [109]. Local reactions—mainly erythema and flaking—were generally mild to moderate, peaking between days 5–8, and resolved spontaneously by day 29. Application site pain (≈8–10%) and pruritus (≈9–11%) were the most common adverse events, typically transient and self-limited. Among complete responders, 27% maintained clearance at one year, consistent with the chronic nature of actinic field damage [110].

The efficacy and tolerability of tirbanibulin have also been demonstrated in larger treatment areas. A Phase 3 safety study treating up to 100 cm^2^ reported a 77.8% reduction in lesion count by day 57, with a tolerability profile similar to that observed in smaller areas and no clinically relevant systemic absorption [110]. These findings support its feasibility for sequential or extended-area treatment in patients with extensive field cancerization.

Real-world studies reinforced the results from clinical trials. In the PROAK study from the United States, clinician-reported clearance (≥75% reduction) exceeded 70% at weeks 8 and 24, with significant improvements in quality of life and high satisfaction scores for convenience and global outcomes [111]. Similar findings were observed in European cohorts, including the KLIR and TIRBASKIN studies, where >80% of patients and clinicians rated cosmetic outcomes as improved, and most expressed a high likelihood of choosing tirbanibulin again if needed [112,113]. Local reactions were mild for the majority of patients, and adherence was excellent.

In conclusion, current evidence indicates that tirbanibulin 1% is an effective, short-course, and well-tolerated field therapy that improves actinic damage and addresses subclinical dysplasia within the cancerization field. While no trials have evaluated primary prevention of NMSC, its capacity to treat AK and modify the cancerization field supports its role as a secondary chemopreventive strategy aimed at reducing progression risk within chronically sun-damaged skin [107,109].

### 3.7. Tretinoin (See Figure 1 for Retinoids)

Tretinoin is discussed here as a topical agent. It is a vitamin A derivate (retinoid), largely used in dermatology as an acne and photoaging treatment. Although preclinical studies suggest retinoid-mediated modulation of carcinogenesis, these findings have not been demonstrated clinically for topical tretinoin; neither has topical tretinoin shown efficacy in skin cancer chemoprevention. A large randomized controlled trial in high-risk patients demonstrated that 0.1% tretinoin did not reduce the incidence of new BCC or SCC compared with vehicle over a prolonged follow-up [58]. Subsequent reviews confirm the absence of preventive benefit for keratinocyte carcinomas and AKs.

In conclusion, tretinoin remains useful for acne and photoaging, but guidelines do not recommend its use for cancer prevention. However, combination regimens with tretinoin show promising preliminary results but lack prospective validation [114].

### 3.8. Comparative Considerations Across Topical and Field Therapies

Although topical and field therapies are primarily indicated for the treatment of actinic keratoses, comparative data are clinically relevant when these agents are used as intermittent secondary chemopreventive strategies in patients with extensive field cancerization. In the randomized head-to-head trial by Jansen et al., which compared four commonly used field-directed treatments on the head, 5% 5-fluorouracil showed the highest sustained clearance rates at 12 months, supporting its role as a reference option when long-term field control is prioritized [80].

From a practical perspective, this greater efficacy must be balanced against the intensity of local inflammatory reactions and the need for adherence to multi-week regimens. Imiquimod provides an immune-mediated approach with good efficacy but generally lower and less durable clearance compared with 5-fluorouracil in direct and indirect comparisons [80]. Photodynamic therapy offers procedure-based treatment with favourable cosmetic outcomes and supervised delivery, although access, cost, and treatment-related pain may limit its use. Diclofenac gel is associated with lower clearance rates but excellent tolerability, making it suitable for patients requiring large-area treatment or minimal inflammation. Tirbanibulin represents a short-course and well-tolerated option that may improve adherence, although long-term comparative and chemopreventive data are still limited.

In conclusion, selection of topical field therapy should be individualized according to expected durability of clearance, tolerability, acceptable downtime, treatment area, likelihood of adherence, patient preference, and access to office-based procedures. Importantly, for all topical agents, evidence for the prevention of invasive keratinocyte carcinoma remains limited and largely based on AK-related surrogate endpoints.

## 4. Other Agents

### 4.1. Vitamin D

**Figure 12:**
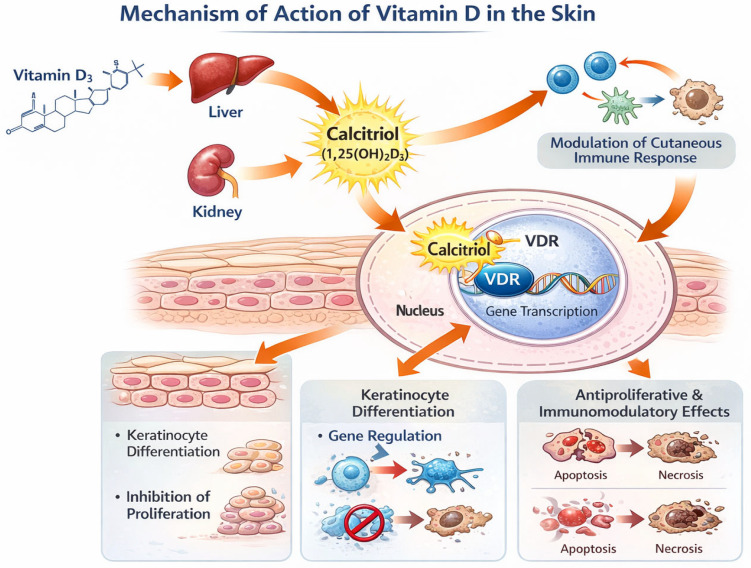
Mechanism of action of vitamin D in the skin. Calcitriol binds to the vitamin D receptor (VDR) and regulates gene transcription, promoting keratinocyte differentiation, inhibiting cell proliferation, and modulating the cutaneous immune response.

Calcitriol, the active form of vitamin D, is involved in cell differentiation, inhibition of proliferation, and interaction with the cutaneous immune system (Figure 12) [115].

The most frequently studied cancers in relation to vitamin D are breast, colorectal, prostate, skin, lung, ovarian, pancreatic, gastric, hepatocellular, thyroid, leukemia, multiple myeloma, bladder, lymphoma, osteosarcoma, cervical, endometrial, and glioblastoma [116]. In the field of skin cancer chemoprevention, dietary supplementation with vitamin D has not demonstrated a reduction in the incidence of skin cancer [117,118]. In contrast, topical formulations of vitamin D derivatives may hold a potential role, although evidence remains limited. Similarly, calcipotriol (a synthetic derivative of calcitriol) may have a synergistic effect with both 5-FU [119] and PDT [120] in the treatment of AKs.

In conclusion, current evidence does not support the role of vitamin D as a chemopreventive agent in either NMSC or melanoma. Clinical trials regarding its use as a chemopreventive agent in skin cancer are needed to draw conclusions.

### 4.2. Polyphenols

**Figure 13:**
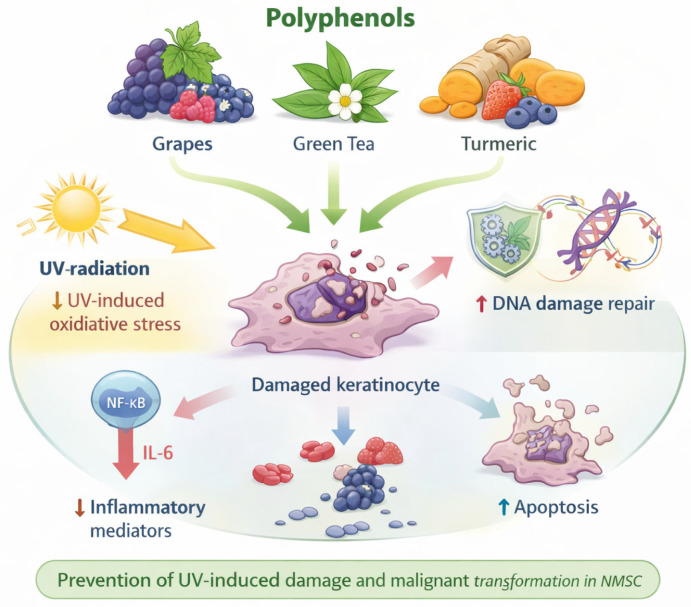
Proposed mechanisms of action of polyphenols in the prevention of non-melanoma skin cancer (NMSC). Polyphenols have antioxidant, anti-inflammatory, and antiproliferative properties that exert chemopreventive effects in NMSC. By reducing UV-induced oxidative stress, enhancing DNA damage repair, and suppressing inflammatory mediators, polyphenols limit UV-mediated keratinocyte damage. In addition, polyphenols promote apoptosis of damaged or dysplastic keratinocytes, thereby preventing malignant transformation and NMSC development.

Polyphenols are phytochemicals with antioxidant, anti-inflammatory, and antiproliferative properties (Figure 13) [121]. In the context of NMSC, polyphenols are of particular interest because they can reduce UV-induced oxidative stress, enhance DNA-damage repair, suppress inflammatory mediators, and promote apoptosis of damaged or dysplastic keratinocytes [121,122]. There are no studies on their efficacy in humans for reducing the number of NMSC, but they seem to be potential candidates for future chemoprevention. Some studied polyphenols include green tea polyphenols (particularly (−)-epigallocatechin-3-gallate [EGCG]), resveratrol, and curcumin. Their main limitation is still their bioavailability and biotransformation.

### 4.3. T4 Endonuclease V

**Figure 14:**
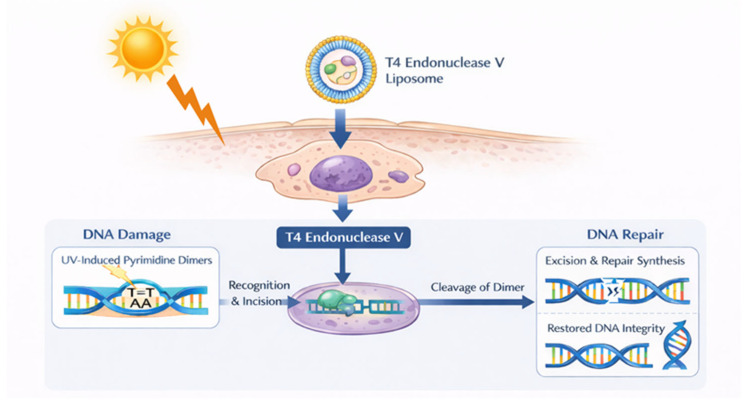
Mechanism of action of the T4 endonuclease V enzyme. The T4 endonuclease V is encapsulated within liposomes and delivered topically to the skin. The enzyme is internalized by keratinocytes into their nuclei where it recognizes, binds, and incises UV-induced pyrimidine dimers on DNA strands.

T4 endonuclease V (T4N5) is a DNA repair enzyme derived from bacteriophage T4 that targets cyclobutane pyrimidine dimers (CPDs), the predominant UV-induced photolesions driving mutagenesis in keratinocytes (Figure 14) [123,124]. From these first observations, its use was suggested for patients with xeroderma pigmentosum [125]. These patients represent a high-risk group for skin cancer as they do not possess the ability to repair UV-induced DNA damage due to inherited mutations in genes encoding proteins that play critical roles in nucleotide excision repair or in the post-replication repair of DNA. The first trial on its efficacy in reducing NMSC was conducted in xeroderma pigmentosum patients by the same group. It was a randomized, placebo-controlled study, with 20 patients in the intervention group and 8 in the placebo group completing the study [126]. The treatment consisted of the application of 4.5 mL of the lotion to the face and arms daily for 1 year. Patients were further followed up at 13 and 18 months. T4N5 liposome lotion lowered the rate of new AKs and BCCs compared with the placebo lotion by 68% and 30%, respectively. The investigators did not observe an increase in the rate of new AK after treatment discontinuation in the 6-month follow-up period. Moreover, they did not observe any adverse events.

Despite these promising results, to date, no other data have been reported on T4N5 use for skin cancer prevention. However, it has recently been proposed as a candidate enzyme for combined or complementary use in sunscreens/after-sun products [127,128].

### 4.4. Ablative Fractional Laser Therapy

**Figure 15:**
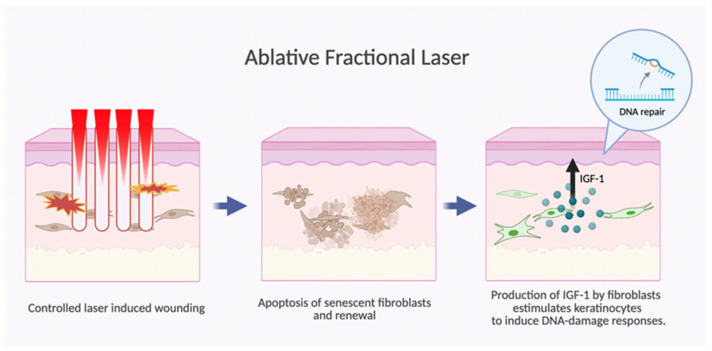
Ablative fractional laser induces fibroblast turnover. Induced wounding eliminates senescent fibroblasts and promotes their turnover. New functionally competent fibroblasts produce IGF-1 that stimulates keratinocytes to initiate an appropriate UV-induced DNA-damage response.

Strictly speaking, laser-based interventions do not fall under the definition of chemoprevention. However, emerging evidence suggests wounding therapies such as laser treatment to be promising in the primary and secondary prevention of NMSC [129]. The underlying rationale is that controlled laser-induced wounding eliminates senescent fibroblasts and promotes fibroblast turnover. Functionally competent fibroblasts are required to produce IGF-1, which has been shown to be essential for keratinocytes to initiate an appropriate UVB-induced DNA-damage response (Figure 15) [130,131].

In a pilot study, each participant received a single ablative fractional resurfacing treatment on one forearm while the contralateral forearm served as an untreated control [132]. A blinded assessment for the presence of AK was performed during follow-up. At 6 months, the treated forearms demonstrated a mean 60% reduction in total AK count, whereas the untreated forearms exhibited a 167% mean increase in AK number [132]. In their subsequent study, they prolonged the follow-up to 3 years and counted the number of AK and NMSC [130]. There was again a decrease in the number of AKs over the treated arms and an increase over the untreated arms. The frequency of NMSCs appearing over the treated arms was 0.19%, 4-fold lower than that of the untreated arms, 0.78% [130].

This is a different strategy, focused on the dermal microenvironment rather than the epidermal cells. A combination of both approaches might provide the best results. Publications exist on the efficacy of combined fractional ablative laser and PDT as a treatment for Aks; however, studies with long-term follow-up to assess their chemopreventive use are lacking.

### 4.5. Metformin

**Figure 16:**
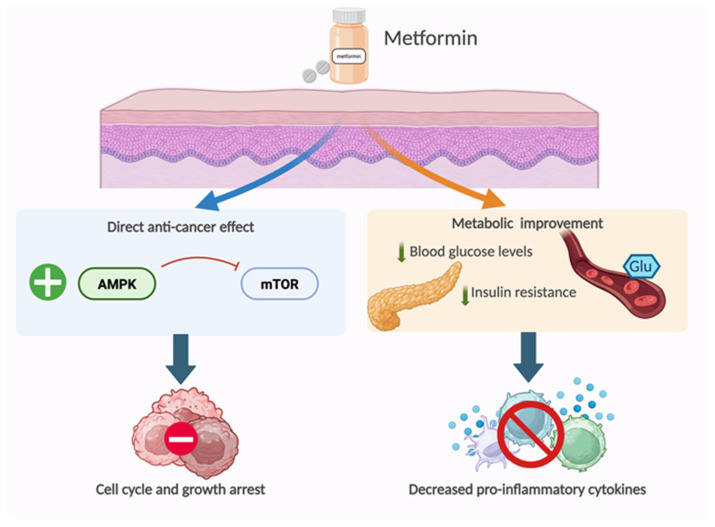
Proposed mechanisms for the potential chemopreventive effect of metformin. Through the activation of the AMPK pathway and subsequent inhibition of mTOR, metformin would modulate cell proliferation and growth. Through the metabolic improvement, it would lead to a decrease in the associated pro-inflammatory state.

Retrospective studies comparing the impact of metformin intake in the incidence of cancer [133], particularly of skin cancer [134,135,136], suggest a moderate preventive effect. The proposed mechanisms by which metformin would prevent cancer occurrence is by reducing inflammation through either (1) the improvement of metabolic disturbances, or (2) by possible anti-inflammatory effects through the inhibition of the Nf-kB, STAT3 and AMPK pathways (Figure 16) [137]. The most recent meta-analysis, however, found no preventive effect of metformin for melanoma or other keratinocyte skin cancers [138]. Most of the included studies do not specifically have the incidence of skin cancer under metformin treatment as an endpoint and are, thus, subject to reporting bias. It is also worth noting that evaluations of the impact of metformin on skin cancer incidence included only diabetic patients [138]. Further prospective studies specifically designed to study a potential chemopreventive effect on skin cancer are needed to draw conclusions.

### 4.6. Carvedilol

**Figure 17:**
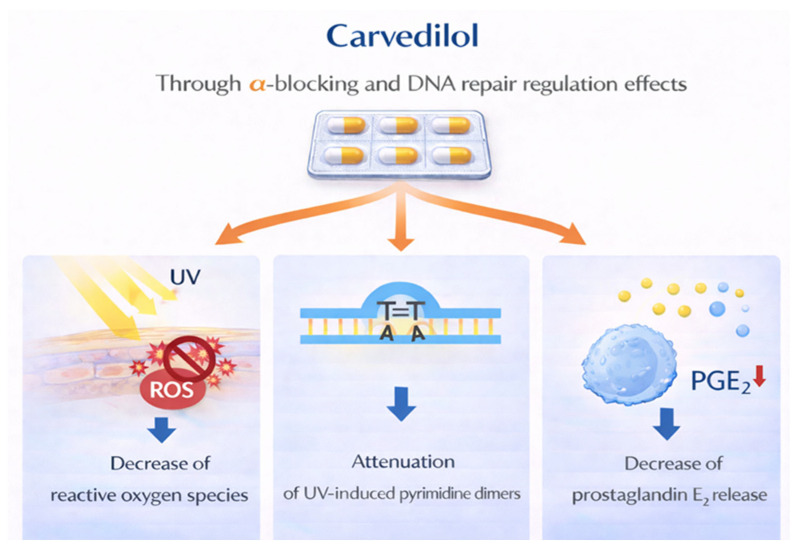
Proposed mechanisms through which carvedilol may act as a chemopreventive agent. Carvedilol acts by decreasing reactive oxygen species (ROS), attenuating UV-induced pyrimidine dimers, and decreasing the release of prostaglandin E2.

Carvedilol is a non-selective β-adrenergic blocker mostly used in chronic heart failure. The anticancer effect of carvedilol would come from an additional α-blocking and DNA repair regulation (Figure 17) [139]. The first data on its preventive effect in skin cancer come from preclinical studies where it was shown that carvedilol, at non-toxic concentrations, inhibited EGF-induced neoplastic transformation of epidermal cells and reduced DMBA-induced epidermal hyperplasia and H-ras mutations in mice [140]. Consequently, it has been proposed as a potential chemopreventive agent for UV-induced skin cancer. New perspectives are being studied and seem promising to bypass the unwanted cardiovascular effects [141,142]. However, to date, there are no studies on its efficacy in humans.

## 5. Photoprotection

Cumulative exposure to UV radiation is the main environmental risk factor for the development of skin cancers, accounting for around 80–90% of cases [143]. Epidemiological evidence consistently links both chronic UV exposure and a history of sunburn—a marker of intense, intermittent UV exposure and individual radiosensitivity—to an increased lifetime risk of skin cancer [144].

Photoprotection includes a broad range of strategies aimed at reducing UV exposure, such as the use of protective clothing, sun avoidance, oral photoprotective agents, and topical sunscreens. The latter represents the cornerstone of modern photoprotection and aims to provide reliable protection against solar erythema, photoaging, and photocarcinogenesis during exposure. Sunscreens combine three principal components: UV filters, emollients, and emulsifiers. Organic filters are aromatic compounds that are able to absorb UV radiation and convert it into lower-energy wavelengths, typically released as heat, whereas inorganic micronized sunscreen filters behave as semiconductor metals that partially absorb UV light [143]. Secondary components, such as photostabilizers, film formers, boosters, and sensorial enhancers, contribute to product stability, uniformity, and cosmetic appeal, which are key to ensuring consistent user compliance. The ideal sunscreen should provide balanced, broad-spectrum protection across at least the UVB and UVA ranges (290 nm–400 nm), mimicking the effect of complete photoprotection achieved through shade or clothing [145].

Regular use of sunscreen has been consistently associated with a reduced risk of developing both melanoma and precancerous lesions, and NMSC. Animal studies conducted during the 1980s and 1990s had already demonstrated that sunscreen application protects against UV-induced photodamage and early events in skin carcinogenesis [146,147].

Regarding NMSC, a randomized controlled trial of 1621 adult Australians found a 38% reduction i SCC incidence among subjects who used daily sunscreen compared with discretionary users, after 8 years of follow-up (HR, 0.62; 95% CI 0.38–0.99). The incidence of BCCs was reduced, but it did not reach statistical significance [148]. Two controlled trials investigating the preventive effect of sunscreen on AKs also found significantly lower rates of new lesion development among participants who used sunscreen regularly compared with controls [149,150]. In particular, Thompson et al. demonstrated that daily application of a sunscreen cream with SPF 17 over one summer in patients aged ≥40 years with 1–30 AKs resulted in a reduction in the mean number of lesions by 0.6 per subject, whereas the mean number increased by 1.0 in the placebo cream group (difference, 1.53; 95% CI, 0.81–2.25). Moreover, the sunscreen group showed a significantly lower incidence of new lesions (rate ratio, 0.62; 95% CI, 0.54–0.71) and a higher rate of lesion remission (odds ratio, 1.53; 95% CI, 1.29–1.80) compared with controls [149]. Furthermore, a study in organ transplant recipients also confirmed that the use of sunscreen over 24 months reduced the development of AKs and SCCs [151].

The cohort from the previously mentioned Australian RCT was followed up for more than a decade to evaluate long-term melanoma incidence. This extended analysis reported that participants who had been randomized to daily sunscreen use during the original 4.5-year trial showed a significantly reduced risk of developing invasive melanoma compared with those assigned to discretionary use (HR 0.27; 95% CI 0.08–0.97). No significant difference was observed for melanoma in situ. This study provided the first randomized evidence supporting the protective role of regular sunscreen use against melanoma in a general population [152].

A meta-analysis of 28 studies and 21,069 melanoma patients reported that ever- vs. never-use of sunscreen was inversely associated with melanoma in hospital-based case–control studies (adjusted OR  =  0.57; 95% CI, 0.37–0.87; *p* value  <  0.001), but this difference was not maintained among population-based case–control studies [153]. Another meta-analysis could not corroborate the effectiveness of sunscreen for preventing either melanoma or non-melanoma skin cancers [154]. However, these analyses were characterized by marked heterogeneity in study design and sunscreen formulations, as well as by substantial methodological limitations, and thus may not reflect the real impact of sunscreens in chemoprevention of skin cancer.

In conclusion, photoprotection has demonstrated a reduction in the risk of developing both melanoma and NMSC and should be proposed systematically to patients at risk of skin cancer, including SOTR.

## 6. Conclusions

Several drugs have been studied as chemopreventive agents. However, since the trial outcomes for most of these agents are inconsistent, conclusive research and guidance on effective measures are still lacking. This is partly due to the difficulties in the design and setup of solid clinical trials. The time of treatment needs to be determined as the effects seem to disappear after discontinuation. This way, long-term follow-up and patient compliance are major limitations. Moreover, data on BCC and melanoma are scarce and discouraging.

Indeed, despite recent advances in diagnosis and treatment, cutaneous melanoma remains a significant public health challenge due to its rising incidence, aggressiveness, and limited preventive strategies. Chemoprevention offers a promising field for reducing melanoma risk. Agents such as aspirin, statins, sulforaphane, vitamin D, and N-acetylcisteine (not discussed) have been studied in clinical trials with conflicting, negative, or inconclusive results [155].

Among the systemic chemopreventive agents reviewed in this chapter, the most studied include retinoids, nicotinamide, and NSAIDS. The retinoids acitretin and isotretinoin require liver monitoring and should be avoided in women of childbearing potential as they are teratogenic. Nicotinamide 500 mg twice a day can be advised for immunocompetent patients with a history of SCC according to the latest European guidelines [34], with caution regarding mucocutaneous infections. Concerning the use of nicotinamide for SOTRs, the latest results do seem to indicate a protective effect but are still not currently recommended. NSAID and COX-2 cardiovascular safety in the long term is a concern, and, to date, results on their efficacy are heterogeneous and, thus, not reliable.

Topical and field therapies for AKs can be considered chemopreventive since the treatment of precancerous lesions may prevent the occurrence of later SCC. However, their long-term efficacy when used intermittently needs to be determined. Once again, conducting conclusive clinical trials on this matter seems extremely difficult. The most established chemopreventive agents include topical 5-FU, imiquimod, PDT, topical diclofenac sodium, and, more recently introduced, tirbanibulin. They are all effective treatments for AK, and their recommendation is based on the clinician’s and patient’s preferences.

SOTRs are known to have an increased risk of skin cancer due to lifelong immunosuppression, and they have been the targeted population of many of the trials on chemoprevention [12,14,15,36,40,41,105,106,156,157,158]. Preventive strategies identified include systemic treatments such as low-dose acitretin, capecitabine, niacinamide, and topical therapies such as 5-fluorouracil and photodynamic therapy [156]. Imiquimod has classically been avoided in these patients but has proven to be safe [75,77].

Photoprotection should always be recommended. However, it does not protect against already existing damage, and its effect in reducing skin cancer incidence remains uncertain. Active follow-up and treatment of precursor lesions is also of utmost importance.

Some molecules discussed in this article, such as T4N5, metformin, carvedilol, or polyphenols, have only been studied in a few publications and may deserve further consideration. Moreover, the synergistic effect of the combination of calcipotriol or ablative laser with topical treatments merits long-term studies.

Limitations of the study include it being a review; therefore, some articles may not have been considered according to the authors’ criteria. Preliminary studies on mouse models and/or small sample sizes might have been disregarded.

## 7. Future Directions

Future directions should focus on uniting efforts to conduct large multicenter clinical trials for molecules showing inconsistent results.

Studies with longer follow-up on chemopreventive agents that have already shown their effectiveness in immunocompetent and immunocompromised patients would help elucidate their real protective effect on skin cancer in the long-term for both populations. Challenges remain to prove their efficacy during and after treatment, optimal dosing, long-term safety, and patient selection.

An ideal chemopreventive agent should not only aim to reduce the risk of skin cancer but also be safe, cost-effective, well-tolerated, easy to use, and available in a standardized form [159].

Fundamental research on the mechanisms of carcinogenesis could help identify new targets for chemoprevention. This would lead to more personalized approaches, regarding not only skin cancer but also patient selection.

Finally, more studies should be conducted on melanoma patients because of its higher aggressiveness. Moreover, research on chemopreventive agents for Merkel cell carcinoma and aggressive cutaneous lymphoma is of utmost importance due to their higher mortality rates.

## Figures and Tables

**Table 1:** Summary of the studies on nicotinamide as chemoprevention for skin cancer. NMSC = Non-melanoma skin cancer; AK = Actinic keratosis; SOTR = Solid organ transplant recipient; BCC = Basal cell carcinoma; SCC = Squamous cell carcinoma.

Reference	Condition	Design	Follow-Up	Intervention	Subjects	Endpoint	Size (n)	Results	Year
Surjana et al. [29]	AK	Phase II randomized double-blind, placebo-controlled	4 months	Oral nicotinamide 500 mg 2/d vs. 1/d vs. placebo	Immunocompetent with >4 AKs	AK count change	76 Study 1 + 2	Significant reduction in AK count in the intervention arm	2012
Chen et al. (ONTRAC) [30]	NMSC	Phase III, randomized, double-blind, placebo-controlled	12 months	Oral nicotinamide 500 mg 2/d vs. placebo	Immunocompetent with ≥2 NMSCs in the prior 5 years	New NMSC incidence; AK counts	386	Reduced new NMSC and AK during treatment; benefit waned after discontinuation; well tolerated	2015
Drago et al. [31]	NMSC	Case–control	6 months	Oral nicotinamide 500 mg 2/d	SOTRs with >1 AK	New NMSC incidence	38	Significant reduction in AK size and no new AKs in the intervention arm; controls worsened, and 7 AKs progressed to SCC	2017
Allen et al. (ONTRANS) [33]	NMSC	Phase III, randomized, double-blind, placebo-controlled	12 months	Oral nicotinamide 500 mg 2/d vs. placebo	SOTRs with history of NMSC	Rate of NMSC and AK	158	No significant reduction in NMSC or AK	2023
Hwang et al. [36]	NMSC	Retrospective cohort	12 and 24 months	Oral nicotinamide 500 mg 2/d	SOTRs with confirmed NMSC at least 1 year before nicotinamide initiation	NMSC incidence 1 year before vs. 1 year and 2 years after nicotinamide intake	47 (1 year follow-up cohort) and 31 (2-year follow-up cohort)	Significant reduction for SCC; non-significant for BCC; reductions maintained in 2-year subset	2025
Breglio et al. [35]	NMSC	Retrospective cohort from the Veterans Affairs Corporate Data Warehouse	No limit	Oral nicotinamide 500 mg 2/d for >30 days vs. matched non-exposed	Veterans; includes SOTR subgroup	Time to next NMSC after exposure to nicotinamide	24,822	Reduction in new NMSC after introduction for immunocompetent patients, particularly for SCC, with the greatest reduction when started after the first skin cancer. Non-significant reduction in SOTR but early use associated with lower SCC	2025

## Data Availability

The original contributions presented in this study are included in the article. Further inquiries can be directed to the corresponding author.
